# Supplementary material for: Drug repurposing for aging research using model organisms
Source: Aging Cell. 2017 Jun 16;16(5):1006–15. doi: 10.1111/acel.12626 (PMC5595691; doi:10.1111/acel.12626)
Supplement: Supplementary file 7 — Data S1 Zip‐Archive of all report cards. [file ACEL-16-1006-s007.zip › RC_GDP.pdf]

GDP

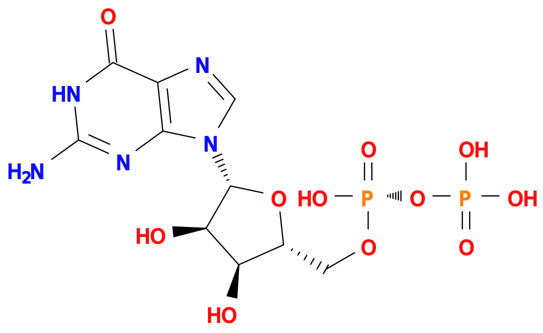

Database identifiers

ChEMBLCompound	CHEMBL384759
CHEBI	17552

Ranking

	Rank	Score
Drosophila	317/697	0.495
C. elegans	578/591	0.0

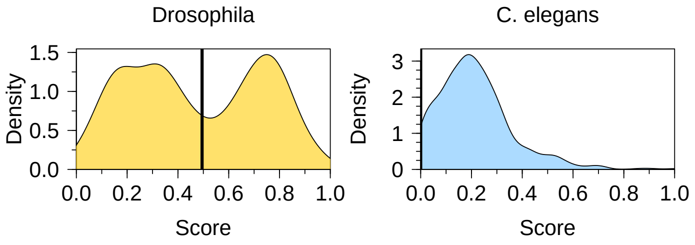

	Ageing implication	Domain conservation	Binding site conservation	Binding affinity	Bioavailability	Lipinski	Promiscuity	Purchasability	Drug approval	**Total**
Drosophila	1.0	0.98	1.0	0.891	(0.9)	-0.1	-0.191	0.0	0.0	0.495
C. elegans	1.0	0.977	1.0	0.891	0.217	-0.1	-0.191	0.0	0.0	0.0

Names

- **GDP**
- Guanosine diphosphate

Roles

ChEBI entry 17552 has no roles

Status

Approved drug (according to ChEMBL)	No
Number of Rule of 5 violations	2
Binding affinity to original target in log units (RF-Score prediction)	7.1
Burns *C. elegans* bioavailability prediction	-4.66

Compound Target Characteristics

GTPase NRas

Best gene implication in ageing for this target family came from gene P01112 annotated in UniProt release 2014_02. Annotation GO subterm of 7568 (aging) was Inferred from Direct Assay

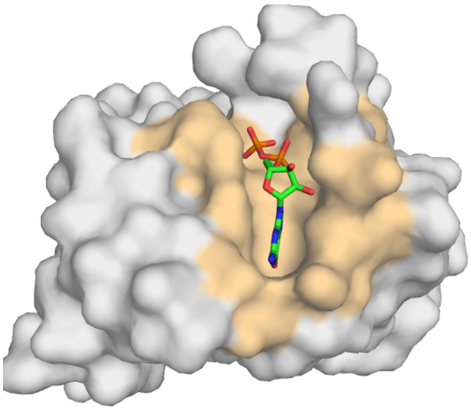

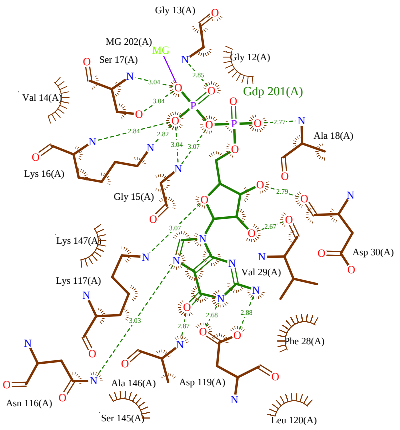

protein	amino acids contacts (binding site)																		
PDB:3con:chainA:P01111	A	G	G	V	G	K	S	A	F	V	D	Y	N	K	D	L	S	A	K
tr:L7RSL8:L7RSL8_HUMAN	A	G	G	V	G	K	S	A	F	V	D	Y	N	K	D	L	S	A	K
sp:P01116:RASK_HUMAN	A	G	G	V	G	K	S	A	F	V	D	Y	N	K	D	L	S	A	K
sp:P01112:RASH_HUMAN	A	G	G	V	G	K	S	A	F	V	D	Y	N	K	D	L	S	A	K
sp:P01111:RASN_HUMAN	A	G	G	V	G	K	S	A	F	V	D	Y	N	K	D	L	S	A	K
tr:Q5U091:Q5U091_HUMAN	A	G	G	V	G	K	S	A	F	V	D	Y	N	K	D	L	S	A	K
tr:I1SRC5:I1SRC5_HUMAN	A	G	G	V	G	K	S	A	F	V	D	Y	N	K	D	L	S	A	K
sp:P20171:RASH_RAT	A	G	G	V	G	K	S	A	F	V	D	Y	N	K	D	L	S	A	K
sp:Q04970:RASN_RAT	A	G	G	V	G	K	S	A	F	V	D	Y	N	K	D	L	S	A	K
sp:P08644:RASK_RAT	A	G	G	V	G	K	S	A	F	V	D	Y	N	K	D	L	S	A	K
tr:A0JN17:A0JN17_RAT	A	G	G	V	G	K	S	A	F	V	D	Y	N	K	D	L	S	A	K
sp:Q61411:RASH_MOUSE	A	G	G	V	G	K	S	A	F	V	D	Y	N	K	D	L	S	A	K
sp:P32883:RASK_MOUSE	A	G	G	V	G	K	S	A	F	V	D	Y	N	K	D	L	S	A	K
tr:Q0VDV7:Q0VDV7_MOUSE	A	G	G	V	G	K	S	A	F	V	D	Y	N	K	D	L	S	A	K
tr:Q5J7N1:Q5J7N1_MOUSE	A	G	G	V	G	K	S	A	F	V	D	Y	N	K	D	L	S	A	K
tr:Q9D091:Q9D091_MOUSE	A	G	G	V	G	K	S	A	F	V	D	Y	N	K	D	L	S	A	K
tr:Q4FJP3:Q4FJP3_MOUSE	A	G	G	V	G	K	S	A	F	V	D	Y	N	K	D	L	S	A	K
sp:P08646:RAS1_DROME	A	G	G	V	G	K	S	A	F	V	D	Y	N	K	D	L	S	A	K
sp:P22981:LET60_CAEEL	D	G	G	V	G	K	S	A	F	V	E	Y	N	K	D	L	S	A	K
sp:P01119:RAS1_YEAST	G	G	G	V	G	K	S	A	F	V	D	Y	N	K	D	L	S	A	K
sp:P01120:RAS2_YEAST	G	G	G	V	G	K	S	A	F	V	D	Y	N	K	D	L	S	A	K

protein	whole protein ident	simil	domain-based ident	simil	contact-based ident	simil
PDB:3con:chainA:P01111	1.0	1.0	1.0	1.0	1.0	1.0
tr:L7RSL8:L7RSL8_HUMAN	0.87	0.96	0.92	0.98	1.0	1.0
sp:P01116:RASK_HUMAN	0.87	0.96	0.92	0.98	1.0	1.0
sp:P01112:RASH_HUMAN	0.85	0.96	0.9	0.98	1.0	1.0
sp:P01111:RASN_HUMAN	1.0	1.0	1.0	1.0	1.0	1.0
tr:Q5U091:Q5U091_HUMAN	1.0	1.0	1.0	1.0	1.0	1.0
tr:I1SRC5:I1SRC5_HUMAN	0.56	0.61	0.92	0.98	1.0	1.0
sp:P20171:RASH_RAT	0.85	0.96	0.9	0.98	1.0	1.0
sp:Q04970:RASN_RAT	0.99	1.0	1.0	1.0	1.0	1.0
sp:P08644:RASK_RAT	0.88	0.96	0.93	0.98	1.0	1.0
tr:A0JN17:A0JN17_RAT	0.85	0.94	0.91	0.97	1.0	1.0
sp:Q61411:RASH_MOUSE	0.85	0.96	0.9	0.98	1.0	1.0
sp:P32883:RASK_MOUSE	0.88	0.96	0.93	0.98	1.0	1.0
tr:Q0VDV7:Q0VDV7_MOUSE	0.88	0.96	0.93	0.98	1.0	1.0
tr:Q5J7N1:Q5J7N1_MOUSE	0.85	0.94	0.91	0.97	1.0	1.0
tr:Q9D091:Q9D091_MOUSE	0.99	1.0	1.0	1.0	1.0	1.0
tr:Q4FJP3:Q4FJP3_MOUSE	0.97	0.98	1.0	1.0	1.0	1.0
sp:P08646:RAS1_DROME	0.75	0.9	0.82	0.94	1.0	1.0
sp:P22981:LET60_CAEEL	0.74	0.88	0.8	0.93	0.89	0.91
sp:P01119:RAS1_YEAST	0.35	0.52	0.62	0.86	0.95	0.97
sp:P01120:RAS2_YEAST	0.34	0.5	0.63	0.87	0.95	0.97

Ras85D (FBgn0003205) associated phenotypes

RU486 conditional, cell autonomous, cell death defective, cell non-autonomous, cell polarity defective, cell size defective, conditional, decreased cell death, decreased cell number, decreased cell size, developmental rate defective, dominant, endocytosis defective, female semi-sterile, germline clone, heat sensitive, hyperplasia, increased cell death, increased cell growth, increased cell number, increased cell size, large body, learning defective, lethal - all die before end of P-stage, lethal - all die before end of pupal stage, majority die during P-stage, maternal effect, melanotic mass phenotype, mitotic cell cycle defective, neoplasia, neuroanatomy defective, neurophysiology defective, partially, partially lethal - majority die, short lived, size defective, small body, somatic clone, some die during P-stage, some die during pharate adult stage, some die during pupal stage, temperature conditional, terminal phenotype, tumorigenic

(Information from FlyBase)

Ras85D (UniProt:P08646) annotation

Function: May mediate a signal that determines the fate of photoreceptor cells in the developing compound eye. Ras proteins bind GDP/GTP and possess intrinsic GTPase activity.

Enzyme regulation: Alternates between an inactive form bound to GDP and an active form bound to GTP. Activated by a guanine nucleotide-exchange factor (GEF) and inactivated by a GTPase- activating protein (GAP).

Subcellular location: Cell membrane (PubMed:18503409); Lipid-anchor (PubMed:18503409); Cytoplasmic side (PubMed:18503409). Note=Loss of prenylation causes protein location to the cytoplasm.

Tissue specificity: Expressed in the posterior termini of the embryo, restricted mainly to the embryonic central nervous system, and in the eye imaginal disk. (PubMed:7873789).

(Information from UniProt)

let-60 (WBGene00002335) associated phenotypes

AWA odorant chemotaxis defective, AWC odorant chemotaxis defective, L2 arrest, L3 arrest, antibody staining reduced, antibody staining variant, axon outgrowth variant, bloated, cell differentiation variant, egg laying defective, egg laying imipramine resistant, egg laying variant, germ cell development variant, gonad arm morphology variant, lethal, locomotion variant, male mating defective, mid larval lethal, multivulva, neuron degeneration, oocyte accumulation, oocytes small, organism starvation response variant, protein expression absent, reduced brood size, rod like larval lethal, sterile, unfertilized oocytes laid, vulval cell induction increased, vulvaless

(Information from WormBase)

let-60 (UniProt:P22981) annotation

Function: The level of let-60 controls the switch between vulval and hypodermal cell fates during C.elegans vulval induction. May stimulate the guanine nucleotide exchange factor (GEF) activity of rap-1.

Subunit: Interacts with soc-2. (PubMed:9674433).

Subcellular location: Cell membrane; Lipid-anchor.

(Information from UniProt)

GTPase HRas

Best gene implication in ageing for this target family came from gene P01112 annotated in UniProt release 2014_02. Annotation GO subterm of 7568 (aging) was Inferred from Direct Assay

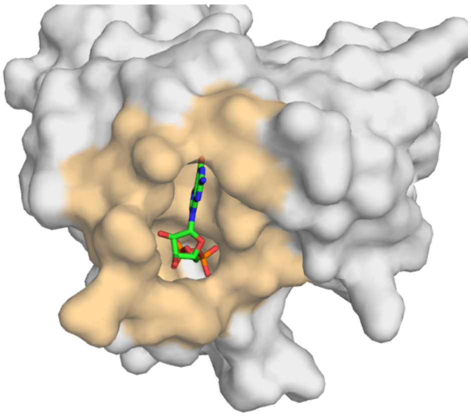

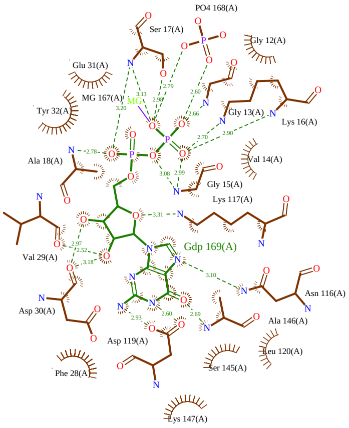

protein	amino acids contacts (binding site)																									
PDB:1aa9:chainA:P01112	A	G	G	V	G	K	S	A	F	V	D	E	Y	D	P	T	A	G	N	K	D	L	T	S	A	K
PDB:1crp:chainA:P01112	A	G	G	V	G	K	S	A	F	V	D	E	Y	D	P	T	A	G	N	K	D	L	T	S	A	K
PDB:1crq:chainA:P01112	A	G	G	V	G	K	S	A	F	V	D	E	Y	D	P	T	A	G	N	K	D	L	T	S	A	K
PDB:1crr:chainA:P01112	A	G	G	V	G	K	S	A	F	V	D	E	Y	D	P	T	A	G	N	K	D	L	T	S	A	K
PDB:1ioz:chainA:P01112	A	G	G	V	G	K	S	A	F	V	D	E	Y	D	P	T	A	G	N	K	D	L	T	S	A	K
PDB:1lf5:chainA:P01112	A	G	G	V	G	K	S	A	F	V	D	E	Y	D	P	T	G	G	N	K	D	L	T	S	A	K
PDB:1pll:chainA:P01112	A	P	G	V	G	K	S	A	F	V	D	E	Y	D	P	T	A	G	N	K	D	L	T	S	A	K
PDB:1q21:chainA:P01112	A	G	G	V	G	K	S	A	F	V	D	E	Y	D	P	T	A	G	N	K	D	L	T	S	A	K
PDB:1xd2:chainA:P01112	A	G	G	V	G	K	S	A	F	V	D	E	Y	D	P	T	A	G	N	K	D	L	T	S	A	K
PDB:1xj0:chainA:P01112	A	G	G	V	G	K	S	A	F	V	D	E	Y	D	P	T	A	A	N	K	D	L	T	S	A	K
PDB:1zvq:chainA:P01112	A	G	G	V	G	K	S	A	F	V	D	E	Y	D	P	T	A	G	N	K	D	L	T	S	A	K
PDB:2ce2:chainX:P01112	A	G	G	V	G	K	S	A	F	V	D	E	C	D	P	T	A	G	N	K	D	L	T	S	A	K
PDB:2cld:chainX:P01112	A	G	G	V	G	K	S	A	F	V	-	-	-	D	P	T	A	G	N	K	D	L	T	S	A	K
PDB:2q21:chainA:P01112	A	V	G	V	G	K	S	A	F	V	D	E	Y	D	P	T	A	G	N	K	D	L	T	S	A	K
PDB:2quz:chainA:P01112	A	G	G	V	G	K	S	A	F	V	D	E	Y	D	P	T	A	G	N	R	D	L	T	S	A	K
PDB:2x1v:chainA:P01112	A	G	G	V	G	K	S	A	F	V	-	-	-	-	-	-	A	-	N	K	D	L	T	S	A	K
PDB:3kud:chainA:P01112	A	G	G	V	G	K	S	A	F	V	D	E	Y	D	P	T	A	G	N	K	D	L	T	S	A	K
PDB:3lo5:chainA:P01112	A	G	G	V	G	K	N	A	F	V	-	-	-	-	-	-	-	-	N	K	D	L	T	S	A	K
PDB:3lo5:chainC:P01112	A	G	G	V	G	K	N	A	-	-	-	-	-	-	-	-	A	G	N	K	D	L	T	S	A	K
PDB:3lo5:chainE:P01112	A	G	G	V	G	K	N	A	F	V	D	E	Y	D	P	T	-	-	N	K	D	L	T	S	A	K
PDB:4l9s:chainA:P01112	A	C	G	V	G	K	S	A	F	V	D	E	Y	D	P	T	A	G	N	K	D	L	T	S	A	K
PDB:4q21:chainA:P01112	A	G	G	V	G	K	S	A	F	V	D	E	Y	D	P	T	A	G	N	K	D	L	T	S	A	K
tr:L7RSL8:L7RSL8_HUMAN	A	G	G	V	G	K	S	A	F	V	D	E	Y	D	P	T	A	G	N	K	D	L	T	S	A	K
sp:P01111:RASN_HUMAN	A	G	G	V	G	K	S	A	F	V	D	E	Y	D	P	T	A	G	N	K	D	L	T	S	A	K
tr:Q5U091:Q5U091_HUMAN	A	G	G	V	G	K	S	A	F	V	D	E	Y	D	P	T	A	G	N	K	D	L	T	S	A	K
sp:P01112:RASH_HUMAN	A	G	G	V	G	K	S	A	F	V	D	E	Y	D	P	T	A	G	N	K	D	L	T	S	A	K
sp:P01116:RASK_HUMAN	A	G	G	V	G	K	S	A	F	V	D	E	Y	D	P	T	A	G	N	K	D	L	T	S	A	K
tr:I1SRC5:I1SRC5_HUMAN	A	G	G	V	G	K	S	A	F	V	D	E	Y	D	P	T	A	G	N	K	D	L	T	S	A	K
sp:P20171:RASH_RAT	A	G	G	V	G	K	S	A	F	V	D	E	Y	D	P	T	A	G	N	K	D	L	T	S	A	K
sp:Q04970:RASN_RAT	A	G	G	V	G	K	S	A	F	V	D	E	Y	D	P	T	A	G	N	K	D	L	T	S	A	K
sp:P08644:RASK_RAT	A	G	G	V	G	K	S	A	F	V	D	E	Y	D	P	T	A	G	N	K	D	L	T	S	A	K
tr:A0JN17:A0JN17_RAT	A	G	G	V	G	K	S	A	F	V	D	E	Y	D	P	T	A	G	N	K	D	L	T	S	A	K
sp:Q61411:RASH_MOUSE	A	G	G	V	G	K	S	A	F	V	D	E	Y	D	P	T	A	G	N	K	D	L	T	S	A	K
sp:P32883:RASK_MOUSE	A	G	G	V	G	K	S	A	F	V	D	E	Y	D	P	T	A	G	N	K	D	L	T	S	A	K
tr:Q0VDV7:Q0VDV7_MOUSE	A	G	G	V	G	K	S	A	F	V	D	E	Y	D	P	T	A	G	N	K	D	L	T	S	A	K
tr:Q5J7N1:Q5J7N1_MOUSE	A	G	G	V	G	K	S	A	F	V	D	E	Y	D	P	T	A	G	N	K	D	L	T	S	A	K
tr:Q9D091:Q9D091_MOUSE	A	G	G	V	G	K	S	A	F	V	D	E	Y	D	P	T	A	G	N	K	D	L	T	S	A	K
tr:Q4FJP3:Q4FJP3_MOUSE	A	G	G	V	G	K	S	A	F	V	D	E	Y	D	P	T	A	G	N	K	D	L	T	S	A	K
sp:P08646:RAS1_DROME	A	G	G	V	G	K	S	A	F	V	D	E	Y	D	P	T	A	G	N	K	D	L	T	S	A	K
sp:P22981:LET60_CAEEL	D	G	G	V	G	K	S	A	F	V	E	E	Y	D	P	T	A	G	N	K	D	L	T	S	A	K
sp:P01119:RAS1_YEAST	G	G	G	V	G	K	S	A	F	V	D	E	Y	D	P	T	A	G	N	K	D	L	T	S	A	K
sp:P01120:RAS2_YEAST	G	G	G	V	G	K	S	A	F	V	D	E	Y	D	P	T	A	G	N	K	D	L	T	S	A	K

protein	whole protein ident	simil	domain-based ident	simil	contact-based ident	simil
PDB:1aa9:chainA:P01112	1.0	1.0	1.0	1.0	1.0	1.0
PDB:1crp:chainA:P01112	1.0	1.0	1.0	1.0	1.0	1.0
PDB:1crq:chainA:P01112	1.0	1.0	1.0	1.0	1.0	1.0
PDB:1crr:chainA:P01112	1.0	1.0	1.0	1.0	1.0	1.0
PDB:1ioz:chainA:P01112	1.0	1.0	1.0	1.0	1.0	1.0
PDB:1lf5:chainA:P01112	0.99	1.0	0.99	1.0	0.96	0.98
PDB:1pll:chainA:P01112	0.99	1.0	0.99	1.0	0.96	0.98
PDB:1q21:chainA:P01112	1.0	1.0	1.0	1.0	1.0	1.0
PDB:1xd2:chainA:P01112	0.99	1.0	0.99	1.0	1.0	1.0
PDB:1xj0:chainA:P01112	0.99	0.99	0.99	0.99	0.96	0.98
PDB:1zvq:chainA:P01112	0.99	1.0	0.99	1.0	1.0	1.0
PDB:2ce2:chainX:P01112	0.99	0.99	0.99	0.99	0.96	0.93
PDB:2cld:chainX:P01112	0.99	1.0	0.99	1.0	0.88	0.77
PDB:2q21:chainA:P01112	0.99	1.0	0.99	1.0	0.96	0.96
PDB:2quz:chainA:P01112	0.99	1.0	0.99	1.0	0.96	0.99
PDB:2x1v:chainA:P01112	0.99	1.0	0.99	1.0	0.73	0.46
PDB:3kud:chainA:P01112	1.0	1.0	1.0	1.0	1.0	1.0
PDB:3lo5:chainA:P01112	0.99	1.0	0.99	1.0	0.65	0.37
PDB:3lo5:chainC:P01112	0.99	1.0	0.99	1.0	0.65	0.37
PDB:3lo5:chainE:P01112	0.99	1.0	0.99	1.0	0.88	0.83
PDB:4l9s:chainA:P01112	0.99	1.0	0.99	1.0	0.96	0.94
PDB:4q21:chainA:P01112	1.0	1.0	1.0	1.0	1.0	1.0
tr:L7RSL8:L7RSL8_HUMAN	0.85	0.95	0.93	0.98	1.0	1.0
sp:P01111:RASN_HUMAN	0.85	0.96	0.9	0.98	1.0	1.0
tr:Q5U091:Q5U091_HUMAN	0.85	0.96	0.9	0.98	1.0	1.0
sp:P01112:RASH_HUMAN	1.0	1.0	1.0	1.0	1.0	1.0
sp:P01116:RASK_HUMAN	0.85	0.95	0.93	0.98	1.0	1.0
tr:I1SRC5:I1SRC5_HUMAN	0.54	0.61	0.93	0.98	1.0	1.0
sp:P20171:RASH_RAT	1.0	1.0	1.0	1.0	1.0	1.0
sp:Q04970:RASN_RAT	0.85	0.96	0.9	0.98	1.0	1.0
sp:P08644:RASK_RAT	0.85	0.95	0.92	0.98	1.0	1.0
tr:A0JN17:A0JN17_RAT	0.84	0.94	0.92	0.98	1.0	1.0
sp:Q61411:RASH_MOUSE	1.0	1.0	1.0	1.0	1.0	1.0
sp:P32883:RASK_MOUSE	0.85	0.95	0.92	0.98	1.0	1.0
tr:Q0VDV7:Q0VDV7_MOUSE	0.85	0.95	0.92	0.98	1.0	1.0
tr:Q5J7N1:Q5J7N1_MOUSE	0.84	0.94	0.92	0.98	1.0	1.0
tr:Q9D091:Q9D091_MOUSE	0.86	0.96	0.9	0.98	1.0	1.0
tr:Q4FJP3:Q4FJP3_MOUSE	0.84	0.94	0.9	0.98	1.0	1.0
sp:P08646:RAS1_DROME	0.77	0.91	0.85	0.95	1.0	1.0
sp:P22981:LET60_CAEEL	0.74	0.89	0.81	0.94	0.92	0.94
sp:P01119:RAS1_YEAST	0.34	0.52	0.61	0.87	0.96	0.98
sp:P01120:RAS2_YEAST	0.34	0.5	0.62	0.88	0.96	0.98

Ras85D (FBgn0003205) associated phenotypes

RU486 conditional, cell autonomous, cell death defective, cell non-autonomous, cell polarity defective, cell size defective, conditional, decreased cell death, decreased cell number, decreased cell size, developmental rate defective, dominant, endocytosis defective, female semi-sterile, germline clone, heat sensitive, hyperplasia, increased cell death, increased cell growth, increased cell number, increased cell size, large body, learning defective, lethal - all die before end of P-stage, lethal - all die before end of pupal stage, majority die during P-stage, maternal effect, melanotic mass phenotype, mitotic cell cycle defective, neoplasia, neuroanatomy defective, neurophysiology defective, partially, partially lethal - majority die, short lived, size defective, small body, somatic clone, some die during P-stage, some die during pharate adult stage, some die during pupal stage, temperature conditional, terminal phenotype, tumorigenic

(Information from FlyBase)

Ras85D (UniProt:P08646) annotation

Function: May mediate a signal that determines the fate of photoreceptor cells in the developing compound eye. Ras proteins bind GDP/GTP and possess intrinsic GTPase activity.

Enzyme regulation: Alternates between an inactive form bound to GDP and an active form

bound to GTP. Activated by a guanine nucleotide-exchange factor (GEF) and inactivated by a GTPase- activating protein (GAP).
Subcellular location: Cell membrane (PubMed:18503409); Lipid-anchor (PubMed:18503409); Cytoplasmic side (PubMed:18503409). Note=Loss of prenylation causes protein location to the cytoplasm.
Tissue specificity: Expressed in the posterior termini of the embryo, restricted mainly to the embryonic central nervous system, and in the eye imaginal disk. (PubMed:7873789).

(Information from UniProt)

let-60 (WBGene00002335) associated phenotypes

AWA odorant chemotaxis defective, AWC odorant chemotaxis defective, L2 arrest, L3 arrest, antibody staining reduced, antibody staining variant, axon outgrowth variant, bloated, cell differentiation variant, egg laying defective, egg laying imipramine resistant, egg laying variant, germ cell development variant, gonad arm morphology variant, lethal, locomotion variant, male mating defective, mid larval lethal, multivulva, neuron degeneration, oocyte accumulation, oocytes small, organism starvation response variant, protein expression absent, reduced brood size, rod like larval lethal, sterile, unfertilized oocytes laid, vulval cell induction increased, vulvaless

(Information from WormBase)

let-60 (UniProt:P22981) annotation

Function: The level of let-60 controls the switch between vulval and hypodermal cell fates during C.elegans vulval induction. May stimulate the guanine nucleotide exchange factor (GEF) activity of rap-1.
Subunit: Interacts with soc-2. (PubMed:9674433).
Subcellular location: Cell membrane; Lipid-anchor.

(Information from UniProt)

GTPase KRas

Best gene implication in ageing for this target family came from gene P01112 annotated in UniProt release 2014_02. Annotation GO subterm of 7568 (aging) was Inferred from Direct Assay

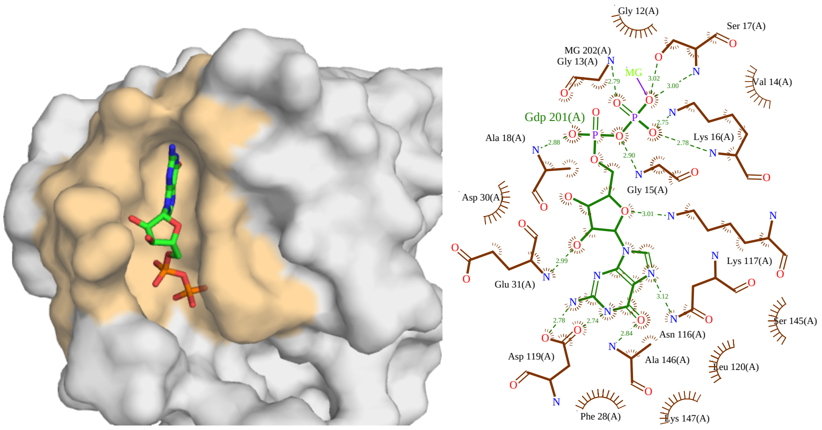

protein	amino acids contacts (binding site)
PDB:4dsu:chainA:P01116	A D G V G K S A F V D E Y A N K D L S A K
PDB:4epr:chainA:P01116	A D G V G K S A F V D E Y A N K D L S A K
PDB:4ept:chainA:P01116	A G G V G K S A F V D E Y A N K D L S A K
PDB:4epv:chainA:P01116	A G G V G K S A F V D E Y A N K D L S A K
PDB:4epw:chainA:P01116	A G G V G K S A F V D E Y A N K D L S A K
PDB:4epx:chainA:P01116	A V G V G K S A F V D E Y A N K D L S A K
PDB:4epy:chainA:P01116	A V G V G K S A F V D E Y A N K D L S A K
PDB:4l8g:chainA:P01116	A C G V G K S A F V D E Y A N K D L S A K
PDB:4ldj:chainA:P01116	A C G V G K S A F V D E Y A N K D L S A K
PDB:4lpk:chainA:P01116	A G G V G K S A F V D E Y A N K D L S A K
PDB:4lpk:chainB:P01116	A G G V G K S A F V D E Y A N K D L S A K
PDB:4lrw:chainA:P01116	A C G V G K S A F V D E Y A N K D L S A K
PDB:4luc:chainA:P01116	A C G V G K S A F V D E Y A N K D L S A K
PDB:4lv6:chainA:P01116	A C G V G K S A F V D E Y A N K D L S A K
PDB:4lyf:chainA:P01116	A C G V G K S A F V D - - A N K D L S A K
PDB:4lyh:chainA:P01116	A C G V G K S A F V D - - A N K D L S A K
PDB:4lyj:chainA:P01116	A C G V G K S A F V D E Y A N K D L S A K
PDB:4m1o:chainA:P01116	A C G V G K S A F V D - - A N K D L S A K
PDB:4m1o:chainC:P01116	A C G V G K S A F V - - - A N K D L S A K
PDB:4m1s:chainA:P01116	A C G V G K S A F V D - - A N K D L S A K
PDB:4m1s:chainC:P01116	A C G V G K S A F - - - - A N K D L S A K
PDB:4m1t:chainA:P01116	A C G V G K S A F V D - - A N K D L S A K
PDB:4m1t:chainC:P01116	A C G V G K S A F V - - - A N K D L S A K
PDB:4m1w:chainA:P01116	A C G V G K S A F V D - - A N K D L S A K
PDB:4m1y:chainA:P01116	A C G V G K S A F V D E - A N K D L S A K
PDB:4m1y:chainC:P01116	A C G V G K S A F V - - - A N K D L S A K
PDB:4m21:chainA:P01116	A C G V G K S A F V D - - A N K D L S A K
PDB:4m21:chainC:P01116	A C G V G K S A F - - - - A N K D L S A K
PDB:4m22:chainA:P01116	A C G V G K S A F V D E Y A N K D L S A K
PDB:4m22:chainB:P01116	A C G V G K S A F V D E Y A N K D L S A K
PDB:4obe:chainA:P01116	A G G V G K S A F V D E Y A N K D L S A K
tr:L7RSL8:L7RSL8_HUMAN	A G G V G K S A F V D E Y A N K D L S A K
sp:P01111:RASN_HUMAN	A G G V G K S A F V D E Y A N K D L S A K
tr:Q5U091:Q5U091_HUMAN	A G G V G K S A F V D E Y A N K D L S A K
sp:P01112:RASH_HUMAN	A G G V G K S A F V D E Y A N K D L S A K
sp:P01116:RASK_HUMAN	A G G V G K S A F V D E Y A N K D L S A K
tr:I1SRC5:I1SRC5_HUMAN	A G G V G K S A F V D E Y A N K D L S A K
sp:P20171:RASH_RAT	A G G V G K S A F V D E Y A N K D L S A K
sp:Q04970:RASN_RAT	A G G V G K S A F V D E Y A N K D L S A K
sp:P08644:RASK_RAT	A G G V G K S A F V D E Y A N K D L S A K
tr:A0JN17:A0JN17_RAT	A G G V G K S A F V D E Y A N K D L S A K
sp:Q61411:RASH_MOUSE	A G G V G K S A F V D E Y A N K D L S A K
sp:P32883:RASK_MOUSE	A G G V G K S A F V D E Y A N K D L S A K
tr:Q0VDV7:Q0VDV7_MOUSE	A G G V G K S A F V D E Y A N K D L S A K
tr:Q5J7N1:Q5J7N1_MOUSE	A G G V G K S A F V D E Y A N K D L S A K
tr:Q9D091:Q9D091_MOUSE	A G G V G K S A F V D E Y A N K D L S A K
tr:Q4FJP3:Q4FJP3_MOUSE	A G G V G K S A F V D E Y A N K D L S A K
sp:P08646:RAS1_DROME	A G G V G K S A F V D E Y A N K D L S A K
sp:P22981:LET60_CAEEL	D G G V G K S A F V E E Y A N K D L S A K
sp:P01119:RAS1_YEAST	G G G V G K S A F V D E Y A N K D L S A K
sp:P01120:RAS2_YEAST	G G G V G K S A F V D E Y A N K D L S A K

protein	whole protein ident	simil	domain-based ident	simil	contact-based ident	simil
PDB:4dsu:chainA:P01116	0.91	0.96	0.95	0.99	1.0	1.0
PDB:4epr:chainA:P01116	0.96	0.99	0.96	0.99	1.0	1.0
PDB:4ept:chainA:P01116	0.97	0.99	0.97	0.99	0.95	0.96
PDB:4epv:chainA:P01116	0.95	0.98	0.96	0.99	0.95	0.96
PDB:4epw:chainA:P01116	0.95	0.98	0.96	0.99	0.95	0.96
PDB:4epx:chainA:P01116	0.95	0.98	0.95	0.98	0.95	0.93
PDB:4epy:chainA:P01116	0.95	0.98	0.95	0.98	0.95	0.93
PDB:4l8g:chainA:P01116	0.96	0.99	0.96	0.99	0.95	0.93
PDB:4ldj:chainA:P01116	0.95	0.98	0.96	0.98	0.95	0.93
PDB:4lpk:chainA:P01116	0.97	0.99	0.97	0.99	0.95	0.96
PDB:4lpk:chainB:P01116	0.98	0.99	0.98	0.99	0.95	0.96
PDB:4lrw:chainA:P01116	0.94	0.97	0.94	0.97	0.95	0.93
PDB:4luc:chainA:P01116	0.94	0.97	0.94	0.97	0.95	0.93
PDB:4lv6:chainA:P01116	0.94	0.97	0.94	0.97	0.95	0.93
PDB:4lyf:chainA:P01116	0.94	0.97	0.94	0.97	0.86	0.73
PDB:4lyh:chainA:P01116	0.94	0.97	0.94	0.97	0.86	0.73
PDB:4lyj:chainA:P01116	0.95	0.97	0.95	0.97	0.95	0.93
PDB:4m1o:chainA:P01116	0.94	0.97	0.94	0.97	0.86	0.73
PDB:4m1o:chainC:P01116	0.94	0.97	0.94	0.97	0.81	0.63
PDB:4m1s:chainA:P01116	0.94	0.97	0.94	0.97	0.86	0.73
PDB:4m1s:chainC:P01116	0.94	0.97	0.94	0.97	0.76	0.53
PDB:4m1t:chainA:P01116	0.94	0.97	0.94	0.97	0.86	0.73
PDB:4m1t:chainC:P01116	0.93	0.97	0.93	0.97	0.81	0.63
PDB:4m1w:chainA:P01116	0.94	0.97	0.94	0.97	0.86	0.73
PDB:4m1y:chainA:P01116	0.94	0.97	0.94	0.97	0.9	0.83
PDB:4m1y:chainC:P01116	0.94	0.97	0.94	0.97	0.81	0.63
PDB:4m21:chainA:P01116	0.94	0.97	0.94	0.97	0.86	0.73
PDB:4m21:chainC:P01116	0.94	0.97	0.94	0.97	0.76	0.53
PDB:4m22:chainA:P01116	0.94	0.97	0.94	0.97	0.95	0.93
PDB:4m22:chainB:P01116	0.95	0.97	0.95	0.97	0.95	0.93
PDB:4obe:chainA:P01116	0.96	0.99	0.96	0.99	0.95	0.96
tr:L7RSL8:L7RSL8_HUMAN	1.0	1.0	1.0	1.0	0.95	0.96
sp:P01111:RASN_HUMAN	0.85	0.95	0.91	0.98	0.95	0.96
tr:Q5U091:Q5U091_HUMAN	0.85	0.95	0.91	0.98	0.95	0.96
sp:P01112:RASH_HUMAN	0.85	0.95	0.91	0.98	0.95	0.96
sp:P01116:RASK_HUMAN	1.0	1.0	1.0	1.0	0.95	0.96
tr:I1SRC5:I1SRC5_HUMAN	0.64	0.64	1.0	1.0	0.95	0.96
sp:P20171:RASH_RAT	0.85	0.95	0.91	0.98	0.95	0.96
sp:Q04970:RASN_RAT	0.86	0.95	0.91	0.98	0.95	0.96
sp:P08644:RASK_RAT	0.99	1.0	0.99	1.0	0.95	0.96
tr:A0JN17:A0JN17_RAT	0.89	0.96	0.94	0.98	0.95	0.96
sp:Q61411:RASH_MOUSE	0.85	0.95	0.91	0.98	0.95	0.96
sp:P32883:RASK_MOUSE	0.99	1.0	0.99	1.0	0.95	0.96
tr:Q0VDV7:Q0VDV7_MOUSE	0.99	1.0	0.99	1.0	0.95	0.96
tr:Q5J7N1:Q5J7N1_MOUSE	0.88	0.95	0.94	0.98	0.95	0.96
tr:Q9D091:Q9D091_MOUSE	0.85	0.95	0.91	0.98	0.95	0.96
tr:Q4FJP3:Q4FJP3_MOUSE	0.83	0.93	0.91	0.98	0.95	0.96
sp:P08646:RAS1_DROME	0.77	0.92	0.81	0.94	0.95	0.96
sp:P22981:LET60_CAEEL	0.74	0.89	0.77	0.93	0.86	0.88
sp:P01119:RAS1_YEAST	0.35	0.52	0.61	0.87	0.9	0.93
sp:P01120:RAS2_YEAST	0.34	0.5	0.61	0.87	0.9	0.93

Ras85D (FBgn0003205) associated phenotypes

RU486 conditional, cell autonomous, cell death defective, cell non-autonomous, cell polarity defective, cell size defective, conditional, decreased cell death, decreased cell number, decreased cell size, developmental rate defective, dominant, endocytosis defective, female semi-sterile, germline clone, heat sensitive, hyperplasia, increased cell death, increased cell growth, increased cell number, increased cell size, large body, learning defective, lethal - all die before end of P-stage, lethal - all die before end of pupal stage, majority die during P-stage, maternal effect, melanotic mass phenotype, mitotic cell cycle defective, neoplasia, neuroanatomy defective, neurophysiology defective, partially, partially lethal - majority die, short lived, size defective, small body, somatic clone, some die during P-stage, some die during pharate adult stage, some die during pupal stage, temperature conditional,

terminal phenotype, tumorigenic

(Information from FlyBase)

Ras85D (UniProt:P08646) annotation
Function: May mediate a signal that determines the fate of photoreceptor cells in the developing compound eye. Ras proteins bind GDP/GTP and possess intrinsic GTPase activity.
Enzyme regulation: Alternates between an inactive form bound to GDP and an active form bound to GTP. Activated by a guanine nucleotide-exchange factor (GEF) and inactivated by a GTPase- activating protein (GAP).
Subcellular location: Cell membrane (PubMed:18503409); Lipid-anchor (PubMed:18503409); Cytoplasmic side (PubMed:18503409). Note=Loss of prenylation causes protein location to the cytoplasm.
Tissue specificity: Expressed in the posterior termini of the embryo, restricted mainly to the embryonic central nervous system, and in the eye imaginal disk. (PubMed:7873789).

(Information from UniProt)

let-60 (WBGene00002335) associated phenotypes
AWA odorant chemotaxis defective, AWC odorant chemotaxis defective, L2 arrest, L3 arrest, antibody staining reduced, antibody staining variant, axon outgrowth variant, bloated, cell differentiation variant, egg laying defective, egg laying imipramine resistant, egg laying variant, germ cell development variant, gonad arm morphology variant, lethal, locomotion variant, male mating defective, mid larval lethal, multivulva, neuron degeneration, oocyte accumulation, oocytes small, organism starvation response variant, protein expression absent, reduced brood size, rod like larval lethal, sterile, unfertilized oocytes laid, vulval cell induction increased, vulvaless

(Information from WormBase)

let-60 (UniProt:P22981) annotation
Function: The level of let-60 controls the switch between vulval and hypodermal cell fates during C.elegans vulval induction. May stimulate the guanine nucleotide exchange factor (GEF) activity of rap-1.
Subunit: Interacts with soc-2. (PubMed:9674433).
Subcellular location: Cell membrane; Lipid-anchor.

(Information from UniProt)

Elongation factor 1-alpha

Best gene implication in ageing for this target family came from gene P08736 annotated in UniProt release 2014_02. Annotation GO 8340 (determination of adult lifespan) was Inferred from Mutant Phenotype

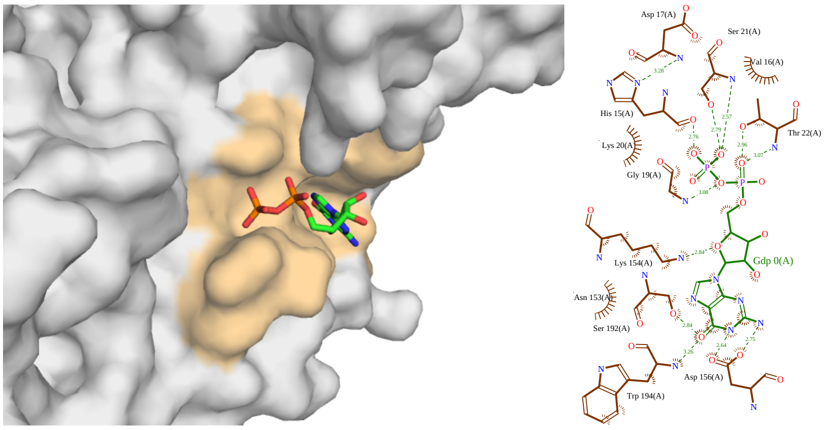

protein	amino acids contacts (binding site)													
PDB:1ije:chainA:P02994	H	V	D	S	G	K	S	T	N	K	D	S	G	W
PDB:1ijf:chainA:P02994	H	V	D	S	G	K	S	T	N	K	D	S	G	W
PDB:2b7b:chainA:P02994	H	V	D	S	G	K	S	T	N	K	D	S	G	W
sp:Q05639:EF1A2_HUMAN	H	V	D	S	G	K	S	T	N	K	D	S	G	W
tr:Q6IPS9:Q6IPS9_HUMAN	H	V	D	S	G	K	S	T	N	K	D	S	G	W
sp:P68104:EF1A1_HUMAN	H	V	D	S	G	K	S	T	N	K	D	S	G	W
tr:D3ZA96:D3ZA96_RAT	H	V	D	S	G	K	S	T	N	K	D	S	G	W
sp:P62630:EF1A1_RAT	H	V	D	S	G	K	S	T	N	K	D	S	G	W
sp:P62632:EF1A2_RAT	H	V	D	S	G	K	S	T	N	K	D	S	G	W
tr:M0R757:M0R757_RAT	H	V	D	S	G	K	S	T	N	K	D	S	G	W
tr:D3YZ68:D3YZ68_MOUSE	H	V	D	S	G	K	S	T	N	K	D	S	G	W
sp:P10126:EF1A1_MOUSE	H	V	D	S	G	K	S	T	N	K	D	S	G	W
sp:P62631:EF1A2_MOUSE	H	V	D	S	G	K	S	T	N	K	D	S	G	W
tr:Q58E64:Q58E64_MOUSE	H	V	D	S	G	K	S	T	N	K	D	S	G	W
sp:P05303:EF1A2_DROME	H	V	D	S	G	K	S	T	N	K	D	S	G	W
tr:A4V3Q6:A4V3Q6_DROME	H	V	D	S	G	K	S	T	N	K	D	S	G	W
tr:C6TP87:C6TP87_DROME	H	V	D	S	G	K	S	T	N	K	D	S	G	W
sp:P08736:EF1A1_DROME	H	V	D	S	G	K	S	T	N	K	D	S	G	W
tr:G8JY74:G8JY74_CAEEL	H	V	D	S	G	K	S	T	N	K	D	S	G	F
sp:P53013:EF1A_CAEEL	H	V	D	S	G	K	S	T	N	K	D	S	G	F
tr:E9P9G3:E9P9G3_YEASX	-	-	-	-	-	-	-	-	N	K	D	S	G	W
sp:P02994:EF1A_YEAST	H	V	D	S	G	K	S	T	N	K	D	S	G	W

protein	whole protein ident	simil	domain-based ident	simil	contact-based ident	simil
PDB:1ije:chainA:P02994	1.0	1.0	1.0	1.0	1.0	1.0
PDB:1ijf:chainA:P02994	1.0	1.0	1.0	1.0	1.0	1.0
PDB:2b7b:chainA:P02994	1.0	1.0	1.0	1.0	1.0	1.0
sp:Q05639:EF1A2_HUMAN	0.79	0.93	0.79	0.94	1.0	1.0
tr:Q6IPS9:Q6IPS9_HUMAN	0.8	0.93	0.79	0.93	1.0	1.0
sp:P68104:EF1A1_HUMAN	0.8	0.93	0.79	0.93	1.0	1.0
tr:D3ZA96:D3ZA96_RAT	0.6	0.8	0.58	0.77	1.0	1.0
sp:P62630:EF1A1_RAT	0.8	0.93	0.79	0.94	1.0	1.0
sp:P62632:EF1A2_RAT	0.79	0.93	0.79	0.94	1.0	1.0
tr:M0R757:M0R757_RAT	0.8	0.93	0.79	0.93	1.0	1.0
tr:D3YZ68:D3YZ68_MOUSE	0.43	0.51	0.79	0.94	1.0	1.0
sp:P10126:EF1A1_MOUSE	0.8	0.93	0.79	0.94	1.0	1.0
sp:P62631:EF1A2_MOUSE	0.79	0.93	0.79	0.94	1.0	1.0
tr:Q58E64:Q58E64_MOUSE	0.8	0.93	0.79	0.94	1.0	1.0
sp:P05303:EF1A2_DROME	0.77	0.93	0.78	0.93	1.0	1.0
tr:A4V3Q6:A4V3Q6_DROME	0.77	0.93	0.78	0.93	1.0	1.0
tr:C6TP87:C6TP87_DROME	0.78	0.93	0.79	0.94	1.0	1.0
sp:P08736:EF1A1_DROME	0.78	0.93	0.79	0.94	1.0	1.0
tr:G8JY74:G8JY74_CAEEL	0.71	0.85	0.67	0.79	0.93	0.97
sp:P53013:EF1A_CAEEL	0.78	0.92	0.79	0.93	0.93	0.97
tr:E9P9G3:E9P9G3_YEASX	0.63	0.63	0.55	0.55	0.43	0.0
sp:P02994:EF1A_YEAST	1.0	1.0	1.0	1.0	1.0	1.0

Ef1alpha100E (FBgn0000557) associated phenotypes
neuroanatomy defective

(Information from FlyBase)

Ef1alpha100E (UniProt:P05303) annotation
Function: This protein promotes the GTP-dependent binding of aminoacyl-tRNA to the A-site of ribosomes during protein biosynthesis.
Subcellular location: Cytoplasm.

(Information from UniProt)

Ef1alpha100E (UniProt:A4V3Q6) annotation
Function: This protein promotes the GTP-dependent binding of aminoacyl-tRNA to the A-site of ribosomes during protein biosynthesis. (RuleBase:RU000325).

(Information from UniProt)

Ef1alpha48D-RA (UniProt:C6TP87) annotation
Function: This protein promotes the GTP-dependent binding of aminoacyl-tRNA to the A-site of ribosomes during protein biosynthesis. (RuleBase:RU000325).

(Information from UniProt)

Ef1alpha48D (FBgn0000556) associated phenotypes
lethal - all die before end of pupal stage, some die during pupal stage

(Information from FlyBase)

Ef1alpha48D (UniProt:P08736) annotation
Function: This protein promotes the GTP-dependent binding of aminoacyl-tRNA to the A-site of ribosomes during protein biosynthesis.
Subcellular location: Cytoplasm.

(Information from UniProt)

eft-3 (UniProt:P53013) annotation
Function: This protein promotes the GTP-dependent binding of aminoacyl-tRNA to the A-site of ribosomes during protein biosynthesis.

Subcellular location: Cytoplasm.

(Information from UniProt)

Phosphoenolpyruvate carboxykinase, cytosolic [GTP]

Best gene implication in ageing for this target family came from gene Q9Z2V4 via mapping the annotation from Ensembl ENSMUSG00000027513 via mapping the annotation from EntrezGene 18534 via mapping the annotation from GenAgeModels 0497 annotated in GenAge release 17.

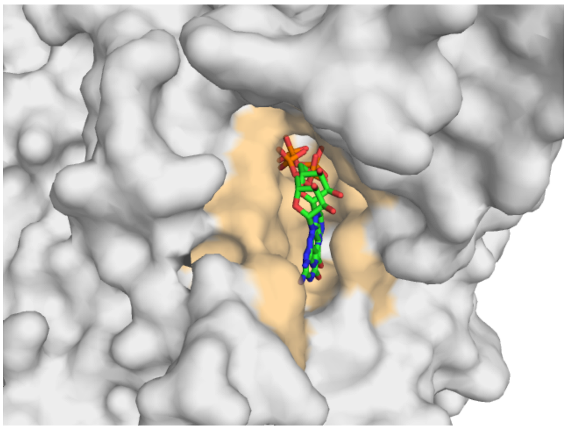

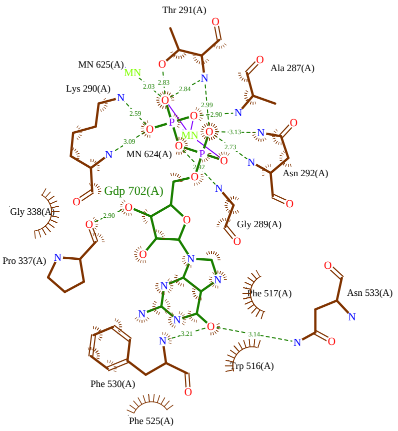

protein	amino acids contacts (binding site)
PDB:2qf2:chainA:P07379	P S A C G K T N V P G R W F F W P G F N
PDB:3dtb:chainA:P07379	P S A C G K T N V P G R W F F W P G F N
PDB:3moh:chainA:P07379	P S A C G K T N V P G R W F F W P G F N
PDB:4gmw:chainA:P07379	P S A C G K T N V P G R W F F W P G F N
PDB:4gnl:chainA:P07379	P S A C G K T N V P G R W F F W P G F N
PDB:4gnp:chainA:P07379	P S A C G K T N V P G R W F F W P G F N
sp:P35558:PCKGC_HUMAN	P S A C G K T N V P G R W F F W P G F N
sp:P07379:PCKGC_RAT	P S A C G K T N V P G R W F F W P G F N
sp:Q9Z2V4:PCKGC_MOUSE	P S A C G K T N V P G R W F F W P G F N
tr:A8DYI3:A8DYI3_DROME	P S A C G K T N V P G R W F F W P G F N
tr:Q7JXB5:Q7JXB5_DROME	P S A C G K T N V P G R W F F W P G F N
tr:Q8ITY1:Q8ITY1_CAEEL	P S A C G K T N V P G R W F F W P G F N
tr:Q8ITY2:Q8ITY2_CAEEL	P S A C G K T N V P G R W F F W P G F N
tr:Q7JKI3:Q7JKI3_CAEEL	P S A C G K T N V P G R W F F W P G F N
tr:Q8ITY0:Q8ITY0_CAEEL	P S A C G K T N V P G R W F F W P G F N
tr:Q7JKI2:Q7JKI2_CAEEL	P S A C G K T N V P G R W F F W P G F N
tr:O44906:O44906_CAEEL	P S A C G K T N V P G R W F F W P G F N
tr:O02286:O02286_CAEEL	P S A C G K T N V P G R W F F W P G F N

protein	whole protein ident	simil	domain-based ident	simil	contact-based ident	simil
PDB:2qf2:chainA:P07379	1.0	1.0	1.0	1.0	1.0	1.0
PDB:3dtb:chainA:P07379	1.0	1.0	1.0	1.0	1.0	1.0
PDB:3moh:chainA:P07379	1.0	1.0	1.0	1.0	1.0	1.0
PDB:4gmw:chainA:P07379	1.0	1.0	1.0	1.0	1.0	1.0
PDB:4gnl:chainA:P07379	1.0	1.0	0.99	1.0	1.0	1.0
PDB:4gnp:chainA:P07379	0.99	1.0	0.99	1.0	1.0	1.0
sp:P35558:PCKGC_HUMAN	0.91	0.98	0.92	0.98	1.0	1.0
sp:P07379:PCKGC_RAT	1.0	1.0	1.0	1.0	1.0	1.0
sp:Q9Z2V4:PCKGC_MOUSE	0.98	0.99	0.97	0.99	1.0	1.0
tr:A8DYI3:A8DYI3_DROME	0.6	0.8	0.7	0.9	1.0	1.0
tr:Q7JXB5:Q7JXB5_DROME	0.62	0.85	0.7	0.9	1.0	1.0
tr:Q8ITY1:Q8ITY1_CAEEL	0.33	0.49	0.56	0.82	1.0	1.0
tr:Q8ITY2:Q8ITY2_CAEEL	0.5	0.73	0.58	0.84	1.0	1.0
tr:Q7JKI3:Q7JKI3_CAEEL	0.55	0.82	0.61	0.87	1.0	1.0
tr:Q8ITY0:Q8ITY0_CAEEL	0.5	0.73	0.51	0.71	1.0	1.0
tr:Q7JKI2:Q7JKI2_CAEEL	0.56	0.84	0.61	0.87	1.0	1.0
tr:O44906:O44906_CAEEL	0.54	0.81	0.58	0.84	1.0	1.0
tr:O02286:O02286_CAEEL	0.54	0.82	0.61	0.87	1.0	1.0

2',3'-cyclic-nucleotide 3'-phosphodiesterase

Best gene implication in ageing for this target family came from gene P13233 annotated in UniProt release 2014_02. Annotation GO 7568 (aging) was Inferred from Expression Pattern

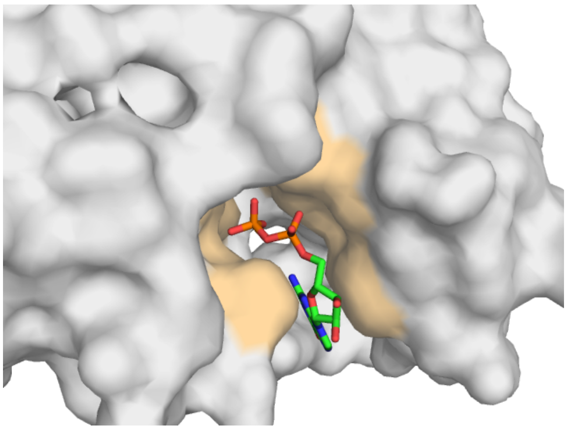

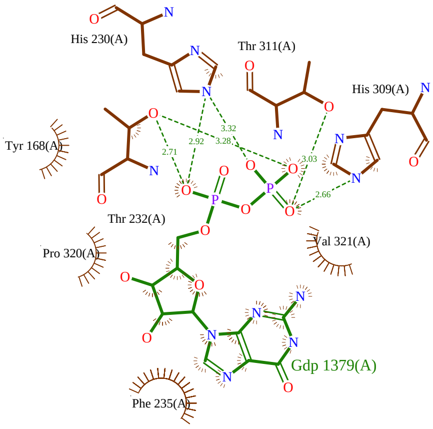

protein	amino acids contacts (binding site)							
PDB:2ydc:chainA:P16330	Y	H	T	F	H	T	P	V
sp:P09543:CN37_HUMAN	Y	H	T	F	H	T	A	V
sp:P13233:CN37_RAT	Y	H	T	F	H	T	P	V
tr:Q3TYL9:Q3TYL9_MOUSE	Y	H	T	F	H	T	P	V
sp:P16330:CN37_MOUSE	Y	H	T	F	H	T	P	V
tr:Q3TYV5:Q3TYV5_MOUSE	Y	H	T	F	H	T	P	V
tr:Q19804:Q19804_CAEEL	-	-	-	-	-	-	-	-

protein	whole protein ident	simil	domain-based ident	simil	contact-based ident	simil
PDB:2ydc:chainA:P16330	1.0	1.0	1.0	1.0	1.0	1.0
sp:P09543:CN37_HUMAN	0.85	0.96	0.8	0.95	0.88	0.97
sp:P13233:CN37_RAT	0.95	0.99	0.95	0.99	1.0	1.0
tr:Q3TYL9:Q3TYL9_MOUSE	0.95	0.95	1.0	1.0	1.0	1.0
sp:P16330:CN37_MOUSE	1.0	1.0	1.0	1.0	1.0	1.0
tr:Q3TYV5:Q3TYV5_MOUSE	1.0	1.0	1.0	1.0	1.0	1.0
tr:Q19804:Q19804_CAEEL	0.11	0.28	0.02	0.05	0.0	0.0

Guanine nucleotide-binding protein G(o) subunit alpha

Best gene implication in ageing for this target family came from gene P59215 annotated in UniProt release 2014_02. Annotation GO 7568 (aging) was Inferred from Direct Assay

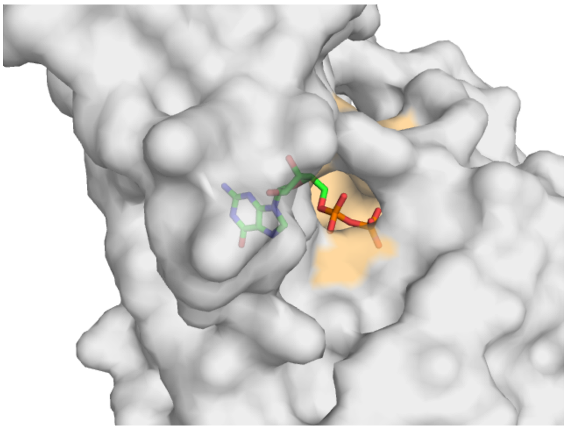

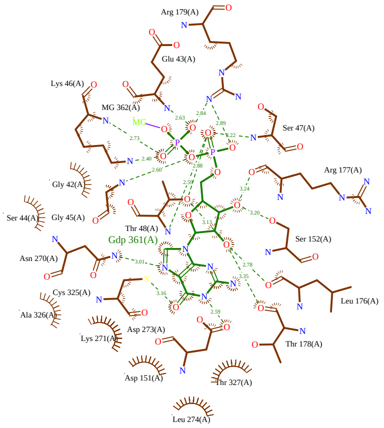

protein	amino acids contacts (binding site)
PDB:3c7k:chainA:P18872	G E S G K S T D S L R T R N K D L C A T
sp:P09471:GNAO_HUMAN	G E S G K S T D S L R T R N K D L C A T
sp:P59215:GNAO_RAT	G E S G K S T D S L R T R N K D L C A T
tr:Q543S2:Q543S2_MOUSE	G E S G K S T D S L R T R N K D L C A T
sp:P18872:GNAO_MOUSE	G E S G K S T D S L R T R N K D L C A T
sp:P16378:GNAO_DROME	G E S G K S T D S L R T R N K D L C A T
sp:P51875:GNAO_CAEEL	G E S G K S T D S L R T R N K D L C A T

	whole protein		domain-based		contact-based	
protein	ident	simil	ident	simil	ident	simil
PDB:3c7k:chainA:P18872	1.0	1.0	1.0	1.0	1.0	1.0
sp:P09471:GNAO_HUMAN	0.98	0.99	0.98	0.99	1.0	1.0
sp:P59215:GNAO_RAT	0.99	1.0	0.99	1.0	1.0	1.0
tr:Q543S2:Q543S2_MOUSE	1.0	1.0	1.0	1.0	1.0	1.0
sp:P18872:GNAO_MOUSE	1.0	1.0	1.0	1.0	1.0	1.0
sp:P16378:GNAO_DROME	0.81	0.94	0.81	0.94	1.0	1.0
sp:P51875:GNAO_CAEEL	0.82	0.94	0.81	0.94	1.0	1.0

Galphao (FBgn0001122) associated phenotypes

cell autonomous, cell non-autonomous, cell polarity defective, circadian rhythm defective, dominant, heat sensitive, mitotic cell cycle defective, neuroanatomy defective, neurophysiology defective, partially lethal - majority die, planar polarity defective, somatic clone, starvation stress response defective, taste perception defective

(Information from FlyBase)

Galphao (UniProt:P16378) annotation

Function: Guanine nucleotide-binding proteins (G proteins) are involved as modulators or transducers in various transmembrane signaling systems. Plays a role in glial cell differentiation during embryogenesis; loco, Galphai and the G-protein coupled receptor, moody, are required in the surface glia to achieve effective insulation of the nerve cord. (PubMed:10079238).

Subunit: G proteins are composed of 3 units; alpha, beta and gamma. The alpha chain contains the guanine nucleotide binding site.

Tissue specificity: Expressed primarily in neuronal cell bodies in the brain, optic lobe, and thoracic and abdominal ganglia. Also expressed in antenna, oocytes and ovarian nurse cells. (PubMed:2509462, PubMed:2509463, PubMed:2509464, PubMed:2519611).

Developmental stage: Expressed in the surface glial cells of the nerve cords at the larval stage (at protein level). Expressed throughout development. (PubMed:10079238, PubMed:2509463).

(Information from UniProt)

goa-1 (WBGene00001648) associated phenotypes

acetylcholine synaptic transmission variant, acetylcholinesterase inhibitor hypersensitive, activity level variant, aldicarb hypersensitive, aldicarb response variant, axon guidance variant, constitutive egg laying, dopamine resistant, early eggs laid, endogenous synaptic events variant, ethanol resistant, halothane resistant, hyperactive, inactive phase variant, inhibition of oocyte maturation variant, isoflurane resistant, levamisole hypersensitive, levamisole response variant, male mating defective, oocyte meiotic maturation variant, pharyngeal pumping reduced, presynaptic component localization variant, spontaneous reversal rate variant

(Information from WormBase)

goa-1 (UniProt:P51875) annotation

Function: Guanine nucleotide-binding proteins (G proteins) are involved as modulators or transducers in various transmembrane signaling systems. The G(o) protein function is not clear. Polarity determinants (par genes) may regulate lin-5/gpr-1/gpr- 2/goa-1 locally to create the asymmetric forces that drive spindle movement (PubMed:12730122). Involved in chemosensory responses to attractive and repellent odors detected by AWC and AWB sensory neurons, respectively (PubMed:23954825). Negatively regulates axon regeneration after injury donwstream of the inhibitory compound arachidonoyl ethanolamide (AEA) by antagonozing the activation of the JNK pathway (mlk-1/mek-1/kgb-1) (PubMed:23072806). (PubMed:12730122, PubMed:23072806, PubMed:23954825).
Subunit: G proteins are composed of 3 units; alpha, beta and gamma. The alpha chain contains the guanine nucleotide binding site. Interacts with gpb-2. When GDP-bound; interacts with gpr-1; gpr-1 forms a complex with gpr-2 and lin-5. (PubMed:12730122).
Disruption phenotype: Prolonged exposure to attractive odorant benzaldehyde results in a loss of adaptive response characterized by a decrease in odor seeking behavior and in a loss of egl-4 nuclear translocation. Loss of chemotaxis response to the repellent odor 2-nonanone. (PubMed:23954825).

(Information from UniProt)

Elongation factor 2

Best gene implication in ageing for this target family came from gene P29691 via mapping the annotation from WormBase WBGene00001167 annotated in WormBase 2014-03-11. Annotation GO 8340 (determination of adult lifespan) was Inferred from Mutant Phenotype

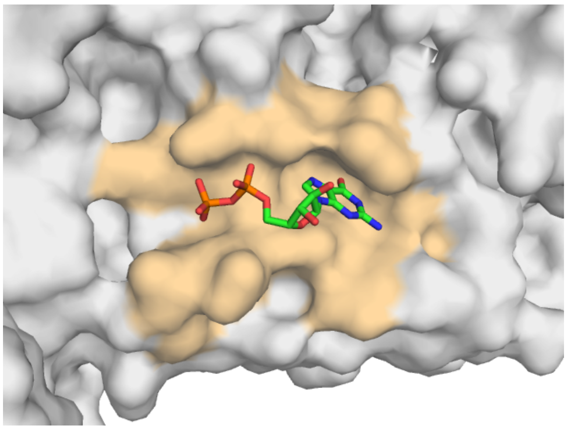

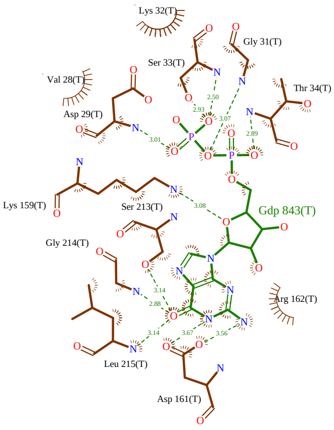

protein	amino acids contacts (binding site)
PDB:1u2r:chainA:P32324	H V D H G K S T N K D R S G L
PDB:2e1r:chainA:P32324	H V D H G K S T N K D R S G L
PDB:2npf:chainA:P32324	H V D H G K S T N K D R S G L
PDB:2p8y:chainT:P32324	H V D H G K S T N K D R S G L
tr:B4DMC6:B4DMC6_HUMAN	H V D H G K S T N K D R S G L
sp:P13639:EF2_HUMAN	H V D H G K S T N K D R S G L
sp:P05197:EF2_RAT	H V D H G K S T N K D R S G L
sp:P58252:EF2_MOUSE	H V D H G K S T N K D R S G L
sp:P13060:EF2_DROME	H V D H G K S T N K D R S G L
sp:P29691:EF2_CAEEL	H V D H G K S T N K D R S G L
sp:P32324:EF2_YEAST	H V D H G K S T N K D R S G L

	whole protein		domain-based		contact-based	
protein	ident	simil	ident	simil	ident	simil
PDB:1u2r:chainA:P32324	1.0	1.0	1.0	1.0	1.0	1.0
PDB:2e1r:chainA:P32324	1.0	1.0	1.0	1.0	1.0	1.0
PDB:2npf:chainA:P32324	1.0	1.0	1.0	1.0	1.0	1.0
PDB:2p8y:chainT:P32324	1.0	1.0	1.0	1.0	1.0	1.0
tr:B4DMC6:B4DMC6_HUMAN	0.29	0.39	0.73	0.91	1.0	1.0
sp:P13639:EF2_HUMAN	0.66	0.88	0.73	0.91	1.0	1.0
sp:P05197:EF2_RAT	0.66	0.88	0.73	0.91	1.0	1.0
sp:P58252:EF2_MOUSE	0.66	0.88	0.73	0.91	1.0	1.0
sp:P13060:EF2_DROME	0.67	0.89	0.7	0.89	1.0	1.0
sp:P29691:EF2_CAEEL	0.67	0.88	0.67	0.86	1.0	1.0
sp:P32324:EF2_YEAST	1.0	1.0	1.0	1.0	1.0	1.0

EF2 (UniProt:P13060) annotation

Function: Catalyzes the GTP-dependent ribosomal translocation step during translation elongation. During this step, the ribosome changes from the pre-translocational (PRE) to the post-translocational (POST) state as the newly formed A-site-bound peptidyl-tRNA and P-site-bound deacylated tRNA move to the P and E sites, respectively. Catalyzes the coordinated movement of the two tRNA molecules, the mRNA and conformational changes in the ribosome (By similarity).
Subcellular location: Cytoplasm
Developmental stage: Expression commences during embryonic germ band elongation and persists throughout development and in the adult. Highest level of expression observed in late embryonic, late larval and early pupal stages. (PubMed:2508059).
Ptm: Phosphorylation by EF-2 kinase completely inactivates EF-2.

(Information from UniProt)

eef-2 (UniProt:P29691) annotation

Function: Catalyzes the GTP-dependent ribosomal translocation step during translation elongation. During this step, the ribosome changes from the pre-translocational (PRE) to the post-translocational (POST) state as the newly formed A-site-bound peptidyl-tRNA and P-site-bound deacylated tRNA move to the P and E sites, respectively. Catalyzes the coordinated movement of the two tRNA molecules, the mRNA and conformational changes in the ribosome. Involved in the morphogenesis of epidermal tissues. (PubMed:18413715).
Subcellular location: Cytoplasm.
Ptm: Phosphorylation by EF-2 kinase completely inactivates EF-2.
Disruption phenotype: Ray defect. (PubMed:18413715).

(Information from UniProt)

Cell division control protein 42 homolog

Best gene implication in ageing for this target family came from gene P60953 via mapping the annotation from Ensembl ENSG00000070831 via mapping the annotation from EntrezGene 998 via mapping the annotation from GenAgeHuman 0250 annotated in GenAge release 17.

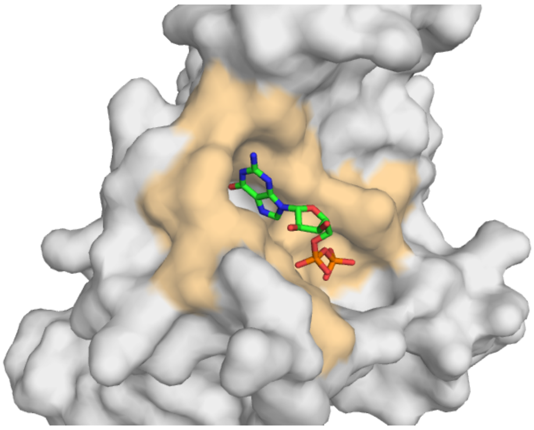

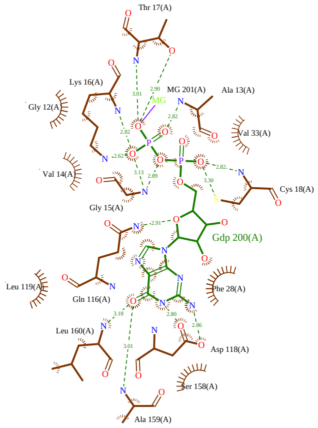

protein	amino acids contacts (binding site)
PDB:1a4r:chainA:P60953	D V A V G K T C F V Q D L S A L
PDB:1an0:chainA:P60953	D G A V G K T C F V Q D L S A L
PDB:1doa:chainA:P60953	D G A V G K T C F V Q D L S A L
PDB:2wmn:chainB:P60953	D G A V G K T C F V Q D L S A L
PDB:3qbv:chainA:P60953	D G A V G K T C F V Q D L S A L
PDB:4did:chainA:P60953	D G A V G K T C F V Q D L S A L
PDB:4itr:chainC:P60953	D G A V G K T C F V Q D L S A L
sp:P60953:CDC42_HUMAN	D G A V G K T C F V Q D L S A L
sp:Q8CFN2:CDC42_RAT	D G A V G K T C F V Q D L S A L
sp:P60766:CDC42_MOUSE	D G A V G K T C F V Q D L S A L
sp:P40793:CDC42_DROME	D G A V G K T C F V Q D L S A L
sp:Q05062:CDC42_CAEEL	D G A V G K T C F V Q D L S A L
sp:P19073:CDC42_YEAST	D G A V G K T C F V Q D L S A L

	whole protein		domain-based		contact-based	
protein	ident	simil	ident	simil	ident	simil
PDB:1a4r:chainA:P60953	0.99	1.0	0.99	1.0	1.0	1.0
PDB:1an0:chainA:P60953	1.0	1.0	1.0	1.0	0.94	0.94
PDB:1doa:chainA:P60953	0.98	0.98	1.0	1.0	0.94	0.94
PDB:2wmn:chainB:P60953	0.99	0.99	1.0	1.0	0.94	0.94
PDB:3qbv:chainA:P60953	0.99	1.0	0.99	1.0	0.94	0.94
PDB:4did:chainA:P60953	1.0	1.0	1.0	1.0	0.94	0.94
PDB:4itr:chainC:P60953	1.0	1.0	1.0	1.0	0.94	0.94
sp:P60953:CDC42_HUMAN	1.0	1.0	1.0	1.0	0.94	0.94
sp:Q8CFN2:CDC42_RAT	1.0	1.0	1.0	1.0	0.94	0.94
sp:P60766:CDC42_MOUSE	1.0	1.0	1.0	1.0	0.94	0.94
sp:P40793:CDC42_DROME	0.93	0.98	0.95	0.99	0.94	0.94
sp:Q05062:CDC42_CAEEL	0.86	0.97	0.9	0.98	0.94	0.94
sp:P19073:CDC42_YEAST	0.8	0.94	0.82	0.95	0.94	0.94

Cdc42 (FBgn0010341) associated phenotypes

cell polarity defective, cell shape defective, chemical resistant, chemical sensitive, decreased cell size, heat sensitive, immune response defective, lethal - all die before end of pupal stage, maternal effect, neuroanatomy defective, neurophysiology defective, non-rescuable maternal effect, partially, partially lethal - majority die, planar polarity defective, size defective, somatic clone, some die during pupal stage, touch sensitivity defective, wound healing defective

(Information from FlyBase)

Cdc42 (UniProt:P40793) annotation

Function: Regulates mbt kinase activity and is also required to recruit mbt to adherens junctions. Together with mbt, regulates photoreceptor cell morphogenesis. (PubMed:12490550).

Subunit: The GTP-bound but not the GDP-bound form interacts with mbt and gek. (PubMed:12490550,

PubMed:9371783).
Subcellular location: Cell junction, adherens junction (PubMed:12490550). Cell membrane (PubMed:12490550); Lipid-anchor (PubMed:12490550). Note=Adherens junctions of developing photoreceptor cells.

(Information from UniProt)

cdc-42 (UniProt:Q05062) annotation

Function: Plays an essential role in spindle orientation and organizing cellular and embryonic polarity by controlling the localization and activity of PAR (partitioning-defective) proteins. Required for maintaining the asymmetric cortical localization of the anterior complex proteins par-3 and par-6, the posterior cortical protein par-2, and pkc-3 (PubMed:11412996, PubMed:11412997). Involved in hypodermal cell fusion, together with pak-1 and ced-10, leading to embryonic body elongation, which involves dramatic cytoskeletal reorganization (PubMed:8824291). During gonad morphogenesis, plays a role in distal tip cell (DTC)- mediated guidance of gonad elongation, probably by activating max- 2 (PubMed:19797046, PubMed:19023419). (PubMed:11412996, PubMed:11412997, PubMed:19023419, PubMed:19797046, PubMed:8824291).
Subunit: The GTP-bound, but not the GDP-bound, form binds to the p21-activated kinase (pak-1). Interaction with par-6 required for activation of the par-3/par-6/pkc-3 complex. (PubMed:11412997, PubMed:8824291).
Subcellular location: Cell membrane (PubMed:8824291); Lipid-anchor ; Cytoplasmic side (PubMed:8824291). Note=Co-localizes with ced-10/rac-1 and pak-1 at hypodermal cell boundaries during embryo elongation (PubMed:8824291).
Tissue specificity: Colocalizes with pak-1 to hypodermal cell boundaries during embryo elongation throughout the second phase of embryogenesis. (PubMed:8824291).
Developmental stage: Highest levels at the embryonic stage, decreasing progressively during development, except for an increase at the L3 stage. (PubMed:8514766).
Disruption phenotype: RNAi-mediated knockdown in distal tip cells (DTC) causes additional turns during their migration. (PubMed:19023419).

(Information from UniProt)

Leucine-rich repeat serine/threonine-protein kinase 2

Best gene implication in ageing for this target family came from gene Q5S007 annotated in UniProt release 2014_02. Annotation GO 8340 (determination of adult lifespan) was Inferred from Mutant Phenotype

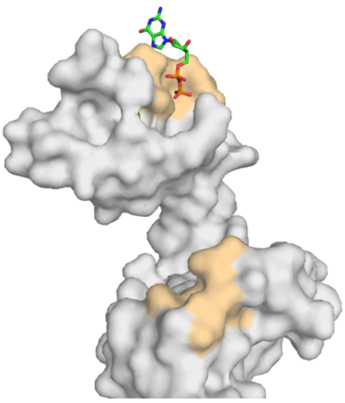

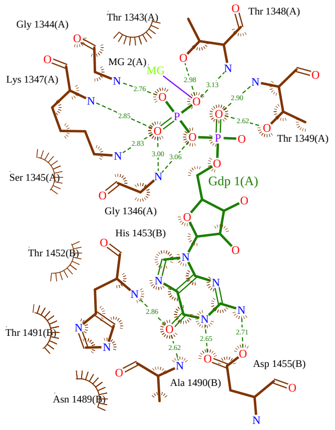

protein	amino acids contacts (binding site)
PDB:2zej:chainA:Q5S007	N T G S G K T T T H D V V N A T
PDB:3d6t:chainB:Q5S007	N T G S G K T T T H D V V N A T
sp:Q5S007:LRRK2_HUMAN	N T G S G K T T T H D V V N A T
tr:F1LNJ1:F1LNJ1_RAT	N T G S G K T T T H D V V N A T
sp:Q5S006:LRRK2_MOUSE	N T G S G K T T T H D V V N A T
tr:Q9VDJ9:Q9VDJ9_DROME	V A G I G K S T T H D A I S C R
sp:Q9TZM3:LRK1_CAEEL	S D G V G K S V T N D Q I N S T

	whole protein		domain-based		contact-based	
protein	ident	simil	ident	simil	ident	simil
PDB:2zej:chainA:Q5S007	1.0	1.0	1.0	1.0	1.0	1.0
PDB:3d6t:chainB:Q5S007	0.95	0.99	0.95	0.99	1.0	1.0
sp:Q5S007:LRRK2_HUMAN	1.0	1.0	1.0	1.0	1.0	1.0
tr:F1LNJ1:F1LNJ1_RAT	0.86	0.96	0.95	0.99	1.0	1.0
sp:Q5S006:LRRK2_MOUSE	0.87	0.96	0.94	0.99	1.0	1.0
tr:Q9VDJ9:Q9VDJ9_DROME	0.15	0.44	0.27	0.6	0.44	0.55
sp:Q9TZM3:LRK1_CAEEL	0.15	0.46	0.27	0.65	0.44	0.63

Lrrk (FBgn0038816) associated phenotypes

chemical resistant, chemical sensitive, female semi-sterile, locomotor behavior defective, neuroanatomy defective, neurophysiology defective, reduced, small body

(Information from FlyBase)

lrk-1 (WBGene00003068) associated phenotypes

pesticide response variant, transgene expression variant

(Information from WormBase)

lrk-1 (UniProt:Q9TZM3) annotation

Function: Determines polarized sorting of synaptic vesicle (SV) proteins to the axons by excluding SV proteins from the dendrite- specific transport machinery in the Golgi. Role in stress response. Appears to antagonize the effects of pink-1 both in the regulation of axon guidance and stress response. (PubMed:17346966, PubMed:19251702).
Cofactor: Mg(2+) Evidence=(UniProtKB:Q38SD2); Name=Mn(2+); Xref=ChEBI:CHEBI:29035; Evidence=(UniProtKB:Q38SD2);
Subcellular location: Golgi apparatus (PubMed:17346966).
Tissue specificity: Expressed in cell bodies, but not in dendritic or axonal processes, of adult head neurons. Also present in non- neuronal tissues, such as the body wall musculature and the epithelial cells of the nematode vulva. (PubMed:17346966, PubMed:19251702).

(Information from UniProt)
